# CARD10 cleavage by MALT1 restricts lung carcinoma growth in vivo

**DOI:** 10.1038/s41389-021-00321-2

**Published:** 2021-04-06

**Authors:** Laura Israël, Anton Glück, Marjorie Berger, Marine Coral, Melanie Ceci, Adeline Unterreiner, Joëlle Rubert, Maureen Bardet, Stefanie Ginster, Alexandra M. Golding-Ochsenbein, Kea Martin, Thomas Hoyler, Thomas Calzascia, Grazyna Wieczorek, Rainer Hillenbrand, Stéphane Ferretti, Enrico Ferrero, Frédéric Bornancin

**Affiliations:** 1 Autoimmunity, Transplantation & Inflammation, Novartis Institutes for BioMedical Research (NIBR), Novartis Campus, Basel, Switzerland; 2 Oncology, NIBR, Novartis Campus, Basel, Switzerland; 3 Chemical Biology & Therapeutics, NIBR, Novartis Campus, Basel, Switzerland; 4 BioMarker Development, Novartis Pharma AG, Novartis Campus, Basel, Switzerland

**Keywords:** Oncogenes, Cell growth

## Abstract

CARD-CC complexes involving BCL10 and MALT1 are major cellular signaling hubs. They govern NF-κB activation through their scaffolding properties as well as MALT1 paracaspase function, which cleaves substrates involved in NF-κB regulation. In human lymphocytes, gain-of-function defects in this pathway lead to lymphoproliferative disorders. CARD10, the prototypical CARD-CC protein in non-hematopoietic cells, is overexpressed in several cancers and has been associated with poor prognosis. However, regulation of CARD10 remains poorly understood. Here, we identified CARD10 as the first MALT1 substrate in non-hematopoietic cells and showed that CARD10 cleavage by MALT1 at R587 dampens its capacity to activate NF-κB. Preventing CARD10 cleavage in the lung tumor A549 cell line increased basal levels of IL-6 and extracellular matrix components in vitro, and led to increased tumor growth in a mouse xenograft model, suggesting that CARD10 cleavage by MALT1 might be a built-in mechanism controlling tumorigenicity.

## Introduction

The NF-κB signaling pathway plays a critical role in several biological processes such as cell proliferation, cell death, or regulation of immune responses^1^. Uncontrolled NF-κB activation in humans leads to autoimmune disorders and cancers. The paracaspase MALT1^2^ is involved in NF-κB signaling cascades downstream of antigen receptors, C-type lectin receptors, and several G-protein coupled receptors^3^. Upon activation, MALT1 scaffolds with BCL10 and a CARD-CC protein^4^ to form a “CBM complex”^5^. The CARD-CC component acts as a seed for assembly of CBM signaling filaments^6^, after a conformational change induced by phosphorylation. Four CARD-CC proteins have been described: CARD11, the most studied, expressed in lymphoid cells, CARD9 expressed in myeloid cells, CARD14 restricted to skin and mucosal tissues, and CARD10 the most broadly expressed in non-hematopoietic cells^7^.

Several genetic mutations associated with human CBM complex components were described. Those resulting in deficiency of either MALT1, BCL10, CARD11, or CARD9 lead to immunodeficiencies displaying increased susceptibility to a wide range of bacterial, viral and/or fungal infections^8^, whereas those resulting in gain-of-function properties in MALT1, CARD9, CARD11, and CARD14 lead to lymphoproliferative or autoimmune disorders^9^. There is currently no evidence for CARD10 germline or somatic mutations in humans. However, CARD10 is overexpressed in many cancers, which has been linked to higher aggressiveness^10–14^.

The CARD10-dependent CBM complex (C_10_BM) signals through protein kinase C (PKC) downstream of G protein-coupled receptors (GPCRs), such as AGTR1 or LPAR^15–17^, and receptor tyrosine kinases (RTKs), such as EGFR^18^. The CARD10 signalosome contributes to NF-κB activation through the regulation of IκB kinase (IκK) complex activity and NF-κB essential modulator (NEMO) polyubiquitination^15^. Deregulation of C_10_BM contributes to tumor growth and poor prognosis^10,18–22^. In most cases, a link could be established between high levels of CARD10 expression and increased cell proliferation, survival, migration, and inflammation triggering angiogenesis and metastasis^13,23^. In addition, C_10_BM was reported to contribute to endothelial dysfunction and vascular disorders^17,20,24^.

The paracaspase activity of MALT1 was discovered in lymphocytes, leading to identification of several substrates, which are best characterized for their modulation of the NF-κB pathway^3^. Here, we identified CARD10 as a novel MALT1 substrate. We showed that cleavage of CARD10 occurs after R587, in the linker region between the coiled-coil and the MAGUK domains, resulting in reduced signaling properties. In a xenograft tumor model using A549 cells injected in nude mice, tumors formed by cells carrying a non-cleavable form of CARD10 were significantly larger than those originating from wild-type (cleavable) CARD10 expressing cells. Broad transcriptomic and proteomic analyses showed that IL-6 and extracellular matrix components are induced when CARD10 cleavage is prevented, suggesting that CARD10 cleavage by MALT1 might be an important mechanism to modulate malignancy.

## Results

### CARD10 is a MALT1 substrate

**Fig. 1:**
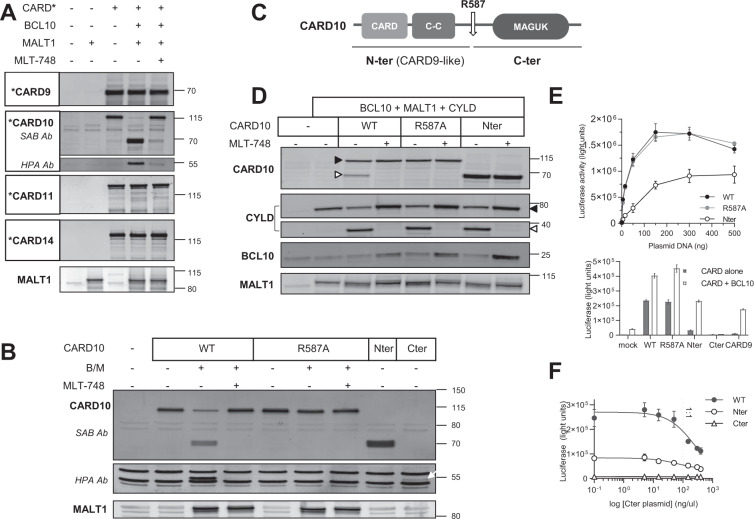
MALT1 cleaves CARD10 at R587. **A** CBM reconstitution assay in HEK 293T cells. Plasmids encoding MALT1, BCL10, and CARD* (either CARD9, CARD10, CARD11, or CARD14) were transfected in HEK 293T cells, alone or in combination, ± 1 µM of MLT-748. Western blot showing the obtained band pattern for each CARD protein. This is one of three experiments with similar results. Plasmids encoding GoF mutant forms of CARD11 and CARD14 were also tested, leading to similar results as those shown here with WT forms. **B** CARD10 constructs were transfected alone or co-transfected with BCL10 and MALT1 (B/M) ± 1 µM of MLT-748. The C-terminal fragment of CARD10 was unstable when overexpressed alone (Fig. 1B, last lane). This is one of three experiments with similar results. **C** Schematic representation of CARD10 including the protein domains and localization of the MALT1-dependent cleavage site at R587. **D** The CARD10 constructs were co-transfected with MALT1 and BCL10 together with the MALT1 substrate CYLD, ± 1 µM of MLT-748. Protein expression and post-activation changes were assessed by Western blot. Full-length proteins are annotated with closed triangles and cleaved fragments with open triangles. Cleaved BCL10 is difficult to detect as it is very similar in size to full length BCL10 and because it is rapidly degraded. Full length BCL10 levels are stabilized by addition of MLT-748, which indirectly accounts for BCL10 cleavage, as demonstrated previously^25^. MALT1 self-cleavage band and mono-ubiquitinated species are visible on the membrane, accounting for stimulation in the absence or presence of a MALT1 inhibitor, respectively, as described previously^25^. This is one of three experiments with similar results. **E** Top, NF-κB reporter luciferase assay after transfection of increasing concentrations of plasmids encoding either CARD10-WT (1–1032) (black filled circles), R587A-CARD10 (grey filled circles), or CARD10-Nter (1–587) (empty circles). Means ± SD of triplicates are shown (*N* = 4); bottom, NF-κB reporter luciferase assay after transfection of CARD10 constructs or CARD9, alone (filled bars) or together with BCL10 (empty bars). Means ± SD of triplicates are shown (*N* = 3). **F** NF-κB reporter luciferase assay upon transfection of CARD10 WT (filled circles), Nter (empty circles), or mock (empty triangles) together with increasing doses of CARD10-Cter plasmid (588–1032); the dotted line indicates the 1:1 dosed vs. tested plasmid ratio. Means ± SD of triplicates are shown (*N* = 4).

Co-expression of CARD-CC proteins^4^ (i.e., CARD9, CARD10, CARD11, or CARD14) in HEK 293T cells, together with MALT1 and BCL10, enables spontaneous assembly and activation of CBM complexes^25^. When this reconstitution assay was performed with CARD10 (C_10_BM), full length CARD10 (115 kD) was readily processed, resulting in two cleavage products of 70 kD and 50 kD (Fig. 1A). Such processing did not occur when C_9_BM, C_11_BM, or C_14_BM were reconstituted. Co-expressing the C_10_BM components in the presence of MLT-748, a potent and selective MALT1 protease inhibitor^26^, abrogated CARD10 cleavage, indicating that the proteolytic function of MALT1, which is induced by CBM assembly, was responsible for CARD10 cleavage under these conditions (Fig. 1A). Thus, CARD10 is a unique CARD-CC protein, sensitive to MALT1 protease within the CBM complex.

MALT1 is an Arginine-directed protease^27,28^. Given the size of the cleaved fragments and the epitopes recognized by the antibodies used, we expected the cleavage site to map around amino acid 600 of CARD10. Using site directed mutagenesis, we therefore replaced individually all Arginine residues located in this region by an Alanine residue (Supplementary Fig. 1A). The R587A mutation was the only one to prevent CARD10 cleavage in the C_10_BM reconstitution assay. This identified R587 as the MALT1-dependent cleavage site, which was corroborated by expression of proteins corresponding to the cleaved fragments, i.e., CARD10_G588X_ (N-ter) and CARD10_588-1032_ (C-ter) (Fig. 1B). The N-terminal CARD10 fragment migrated as a doublet, raising the possibility of a second cleavage event. Further tests ruled out this hypothesis by showing that the upper band is likely a phospho-CARD10 species, also detectable within full length CARD10, or when the N-terminal fragment is expressed alone (Supplementary Fig. 1B, C).

Together, the data indicated that MALT1, when assembled into a C_10_BM complex, is able to cleave CARD10 after R587. The sequence surrounding the R587 site in CARD10 is not conserved in other CARD-CC proteins, consistent with the selective cleavage of CARD10.

From a biochemical standpoint, R587 lies within a LLAR/G P2-to-P4 motif, which matches the preferred substrate specificity motif of MALT1^29,30^. In fact, the P2 Alanine residue in the CARD10 cleavage site is the first example of its occurrence in a MALT1 protein substrate (Supplementary Fig. 2A). From an evolution perspective, analysis of the four CARD-CC isoforms found in jawed vertebrates indicated that the CARD10 cleavage site was absent in reptiles and birds. It appears to be a relatively recent “invention” specific to mammals (Supplementary Fig. 2B).

### Cleavage of CARD10 regulates scaffold-dependent NF-κB activation

CARD10 (as well as CARD11 and CARD14) is composed of a scaffolding CARD domain allowing for interaction with BCL10 and MALT1, a coiled-coil (CC) domain required for oligomerization, which typifies this subfamily of CARD proteins^4^, and a membrane-associated guanylate kinase (MAGUK) domain important for activity^31^ (Fig. 1C). Remarkably, R587 is located in the linker region of CARD10, which connects the N-terminal region (CARD and CC domains) to the C-terminal MAGUK domain. As a result, cleavage of CARD10 by MALT1 would release an N-terminal fragment reminiscent of CARD9, a member of the CARD-CC family that is constitutively devoid of the MAGUK domain.

We first characterized the impact of CARD10 cleavage on CBM-dependent MALT1 proteolytic activation. In the CBM, both BCL10^28^ and MALT1^25,32^ (self-cleavage) are MALT1 protease substrates. In addition to CBM components, we co-transfected CYLD, a MALT1 substrate^33^ previously validated in reconstitution assays^25^. We compared the capacity of WT-CARD10, R587A-CARD10, and N-ter-CARD10 (1-588X) for their ability to trigger the cleavage of each MALT1 substrate (Fig. 1D). Preventing CARD10 cleavage had no effect on BCL10 cleavage, MALT1 C-terminal self-cleavage, and CYLD cleavage. Similarly, the N-ter-CARD10 fragment supported the cleavage of all these substrates. Therefore, the data suggested that cleavage of CARD10 by MALT1 is neither a prerequisite nor an inhibitory event per se for triggering MALT1 proteolytic function.

The CBM complex provides an essential scaffold for recruitment of NF-κB pathway activators such as TNF receptor associated factors (TRAFs) and IκKs^34^. We therefore compared CARD10 and N-ter-CARD10 for their ability to activate NF-κB, using a luciferase reporter gene assay in HEK 293T cells. WT-CARD10 or R587A-CARD10 induced 4.5-fold more luciferase activity than N-ter-CARD10 at low plasmid concentrations, and ~1.5-fold at the highest concentrations tested (Fig. 1E top panel). Of note, CARD9, when tested in this experimental setting also led to weak stimulation of the NF-κB reporter (Fig. 1E bottom panel), but this was strongly enhanced (17-fold change) by co-expression of BCL10, in line with published observations^35^. Similarly, the weaker activity of N-ter-CARD10 by comparison to WT-CARD10 was sensitive to BCL10 co-expression (7-fold change). C-ter-CARD10 expression failed to induce basal NF-κB reporter activity, confirming previous observations with a similar construct^36^, and was insensitive to BCL10 co-expression (Fig. 1E bottom panel). In fact, the C-ter-CARD10 fragment inhibited NF-κB activation in a concentration-dependent manner, when co-expressed with either WT- or N-ter-CARD10 (Fig. 1F). Additional experiments showed that cleavage of CARD10 results in cytoplasmic redistribution of N-ter-CARD10, which is consistent with removal of the membrane anchoring MAGUK domain (Supplementary Fig. 3). These results collectively suggested that cleavage of CARD10 by MALT1 would reduce CARD10-dependent signaling at the membrane while generating a soluble N-ter-CARD10 fragment with lower activity.

### PKC activation triggers MALT1-dependent CARD10 cleavage

**Fig. 2:**
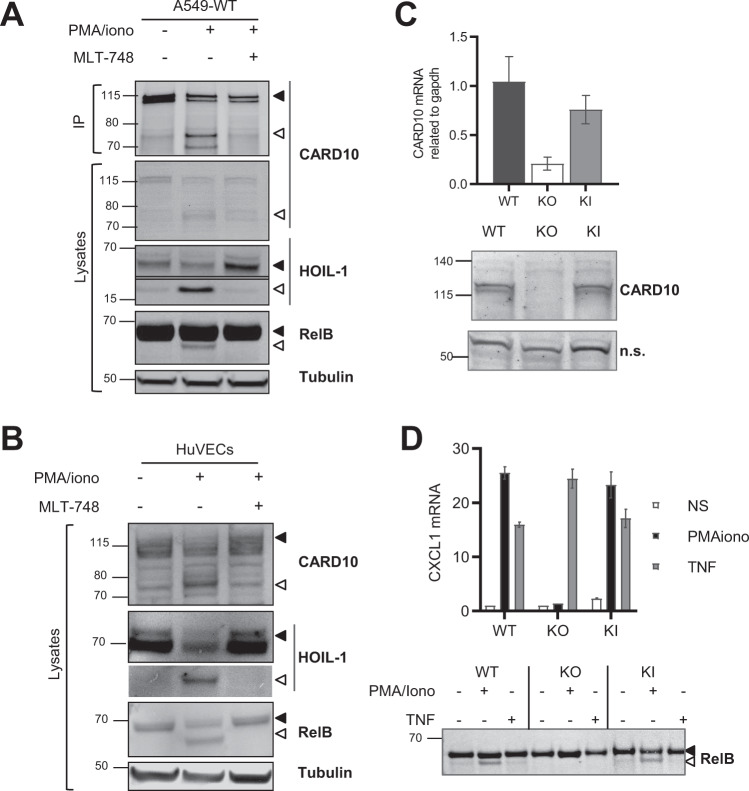
Endogenous CARD10 cleavage in A549 cells and primary HuVEC cells. **A** A549 cells were treated with 5 µM of the proteasome inhibitor MG-132 and stimulated for 2 h with P/I ± 1 µM of MLT-748. CARD10 immunoprecipitation was performed and CARD10 expression assessed by Western blotting together with the two MALT1 substrates HOIL1 and RelB (*N* = 3). **B** HuVECs were treated and analyzed as done in (**A**) for A549 (*N* = 2). **C** CARD10 mRNA levels obtained from microarray analysis with probe 210027 (top panel). CARD10 immunoblot performed on full lysates obtained from A549-WT, -KO, and -KI cells (bottom panel). **D** A549-WT and KI cell lines were treated for 2 h with P/I (50 µg/ml /1 μM) or TNF (10 ng/ml) as non-CBM activation control in presence of 5 μM of MG-132. Relative quantitative PCR measurement of CXCL1 mRNA; Mean ± SD of triplicates (*N* = 2). RelB cleavage was then assessed by Western blot (bottom panel).

Several lines of evidence indicate that PKC mediated phosphorylation of CARD-CC proteins acts as a trigger for CBM assembly downstream of various receptors. In T and B lymphocytes, PKCθ and PKCβ are believed to be the key respective isoforms for activation of CARD11 downstream of antigen receptors^37,38^. In myeloid cells, PKCδ was suggested to carry out phosphorylation of CARD9 downstream of C-type lectin receptors^39^. Similarly, CARD10 and CARD14 are triggered after PKC activation by phorbol esters and possible responsible isoforms have been proposed^15,40,41^. Our laboratory previously reported the PKC-dependency of CARD10 activation in keratinocytes^42^. To provide evidence for endogenous CARD10 cleavage by MALT1, we used A549 human lung carcinoma cells, which express detectable CARD10 levels^10,43^, and we immunoprecipitated CARD10 following phorbol ester/ionomycin treatment (P/I) to activate PKC isoforms^44^. A CARD10 immunoreactive band of ~70 kD was up-regulated by P/I treatment, which was abrogated when carried out in the presence of the MALT1 inhibitor MLT-748 (Fig. 2A). This observation suggested that PKC-dependent CBM complex activation results in MALT1-dependent CARD10 cleavage in A549 cells. Activation of MALT1 paracaspase under these experimental conditions was further evidenced by the cleavage of two known MALT1 substrates, HOIL1^45^ and RelB^46^ (Fig. 2A). Beyond A549 cells, we tested HuVEC primary endothelial cells, a cell type where CARD10 is also known to be expressed^47^, and we similarly observed CARD10 cleavage by MALT1 as well as HOIL-1 and RelB cleavages after P/I treatment (Fig. 2B).

We used the CRISPR-Cas9 method to generate cell lines (A549-KI) expressing the cleavage deficient R587A CARD10 variant form and analyzed them together with a A549-CARD10 KO line generated in parallel. A549-KI and A549-WT clones displayed similar amounts of CARD10, at both mRNA and protein levels. By contrast the CARD10 immunoreactive band was absent in A549-KO cells, validating the CARD10 specific signal despite the limited performance of the anti-CARD10 antibody (Fig. 2C). In addition, C_10_BM-dependent signaling was functional to similar levels in A549-KI and A549-WT clones but abrogated in A549-KO cells, as monitored by CXCL1 mRNA upregulation and RelB cleavage following P/I stimulation. By contrast, TNF-induced CXCL-1, which is independent of C_10_BM, was intact in A549-KI, A549-WT as well as A549-KO cells (Fig. 2D).

### Preventing CARD10 cleavage at R587 accelerates tumor growth

**Fig. 3:**
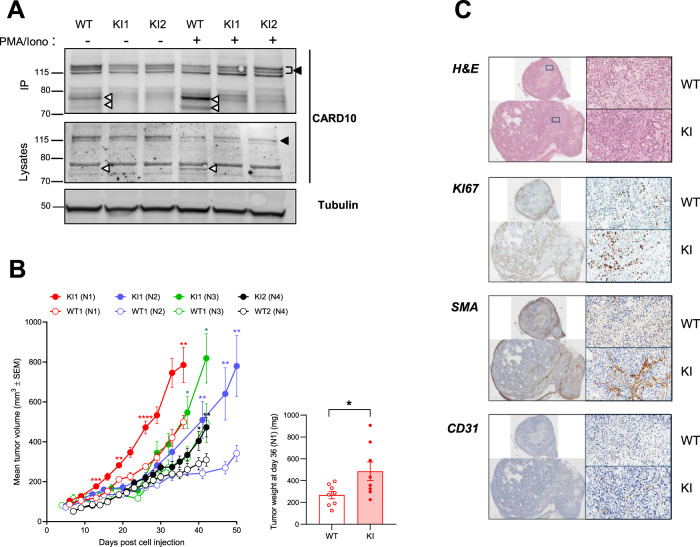
CARD10 cleavage at R587 controls A549 tumor growth. **A** A549 cells (parental WT) or A549-KI (two clones containing the CARD10 point mutation R587A introduced by CRISPR) were treated, stimulated and analyzed for CARD10 as in Fig. 2A. Full length CARD10 and the cleaved CARD10 fragment (both as doublet resulting from differential phosphorylation) are indicated with arrowheads (*N* = 2). **B** 2x10E6 A549-WT cells or KI cells were injected into the flanks of nude mice. Tumor growth was monitored over time and is shown as mean tumor volume (mm^3^) ± SEM (*n* = 8 per group for N1, *n* = 11 for N2, *n* = 6 for N3, and *n* = 8 for N4), which was measured biweekly for 36–50 days. The graph shows the data from four experiments performed independently with the two A549-KI clones. At day 36 (N1 experiment), tumors were extracted and weighed (right panel). Data are reported as means ± SEM. Statistical analyses were carried out using GraphPad Prism software. Differences among groups were assessed using one-tailed Student’s *t* tests. *P* < 0.05 was considered to be statistically significant. **C** Histological analysis of the three biggest tumors per group (N2, described in B) was performed and the data for one representative tumor per group are shown. Tumor sections were stained with H&E for general structure, Ki67 for proliferation, smooth muscle actin (SMA) for stromal cell visualization, and msCD31 for endothelial cell content. The scale is indicated at the bottom left of every image (1 mm for full tumors and 100 µm for the 10X magnification).

Two A549-KI clones (KI1 and KI2) were selected for further evaluation in vivo. By comparison with A549-WT cells, CARD10 cleavage was abrogated in both KI clones, establishing that CBM-induced cleavage of CARD10 at R587 can occur endogenously (Fig. 3A). Furthermore, even though CARD10 cleavage was enhanced upon P/I treatment, it was also detected in the absence of stimulation, suggesting constitutive cleavage of CARD10. Both constitutive and stimulated CARD10 cleavage were abolished by mutation of the R587 MALT1 cleavage site (Fig. 3A).

CARD10 is known to be overexpressed in solid tumors^22^. In mice, A549 cells expressing CARD10 shRNAs are less able to colonize the lung after i.v. injection, as compared to unmodified cells^43^. Given the diminished signaling capacity following CARD10 cleavage shown above, we hypothesized that prevention of CARD10 cleavage might increase its tumorigenic potential. To test this hypothesis the two A549-KI clones were injected in nude mice, in parallel to respective A549-WT control clones, and tumor growth was monitored over time. After a lag period of variable duration across experiments, A549-KI cells, expressing the non-cleavable CARD10 mutant form, started to grow steadily and faster than A549-WT cells. They displayed comparatively a 1.5–2.3 fold increase in volume (Fig. 3B). At termination, tumor weights obtained with KI cells were 1.8 fold heavier on average (*p* = 0.0163, day 36 of N1 experiment) than those obtained with WT cells (Fig. 3B right panel). Analysis of tumors by flow cytometry revealed no significant differences in mouse immune cell infiltration, macrophage numbers, or M1/M2 ratios (Supplementary Fig. 4). However, histopathological analyses revealed the presence of necrotic areas and cell debris in the core of WT tumors whereas those areas were homogenously cellular in KI tumors (Fig. 3C). Ki67 immunostaining showed a homogeneous pattern of proliferating cells in KI tumors whereas proliferation was limited to the periphery of WT tumors (Fig. 3C). In addition, tumor stroma containing smooth muscle actin (SMA)-positive myofibroblasts and CD31-positive endothelial cells was increased in KI tumors as compared to WT tumors (Fig. 3C). We observed vascularization mainly at the periphery of WT tumors. The diminished capillary network could be correlated to necrosis of the core region as observed on H&E stained sections. By contrast, KI tumors were homogeneously vascularized and we noted the formation of larger blood vessels. Collectively, this model showed that the non-cleavable CARD10 mutant form afforded A549 cells with a growth advantage over wild-type cells in vivo.

### CARD10 cleavage regulates extracellular matrix components

Knocking down CARD10 in cancer cells impairs proliferation, survival, and migration^19^. However, how CARD10 contributes to cancer processes has remained ill defined. Prompted by the xenograft model data obtained above, we started by comparing the proliferation rates of A549-WT and KI cells in vitro, their sensitivity to apoptosis, and their migration capacity. None of these read outs, however, revealed notable differences between A549-KI and WT cells (Supplementary Fig. 5). This suggested that CARD10 cleavage might not contribute to tumor growth through cell intrinsic mechanisms.

**Fig. 4:**
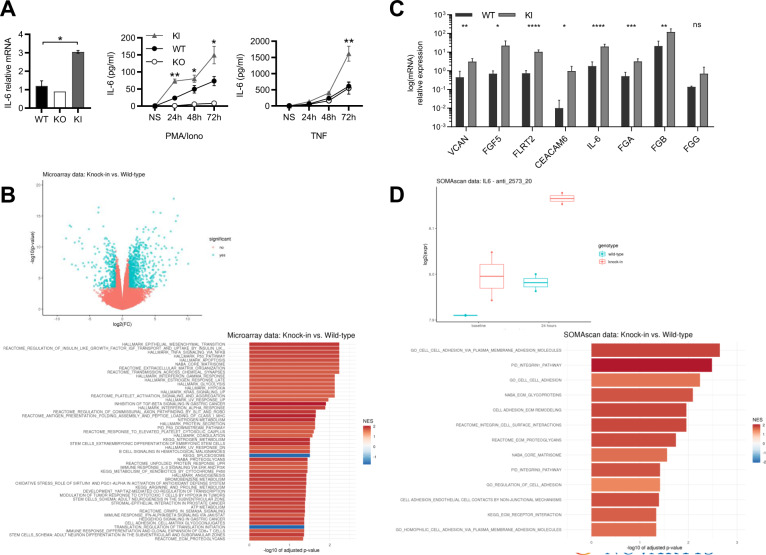
CARD10 cleavage impacts IL-6 and extracellular matrix components. **A** IL-6 mRNA levels measured by RT-PCR (left), 72h-time course of released IL-6 protein levels measured upon P/I (50 µg/ml/1 μM) (middle) or TNF (10 ng/ml) (right) stimulation. Data represent means ± SD of triplicates for two clones of each cell line (*N* = 3). Statistical analyses were carried out using GraphPad Prism software. Differences among groups were assessed using one-tailed Student’s *t* tests. *P* < 0.05 was considered to be statistically significant. **B** Upper panel, Volcano plot of differential mRNA expression in A549-WT and KI cells; microarray probe sets with an absolute log2 fold change of ≥ 1 and a false discovery rate-adjusted *p* ≤ 0.01 are colored in blue. Lower panel, Gene set enrichment analysis of the data shown in the upper panel. Shown are the top 50 of 69 gene sets significant at a false discovery rate < 0.05. *NES* Normalized Enrichment Score. Highly significant pathways were (i) cancer (p53, KRAS) (ii) inflammation (NF-κB, TNF), (iii) cancer-related processes (extracellular matrix (ECM), apoptosis, and angiogenesis), with a particular enrichment of gene sets linked to ECM regulation (matrisome, ECM organization, proteoglycans, cell adhesion-matrix glycogonjugates, and ECM proteoglycans). **C** Relative quantitative PCR measurement of eight selected mRNAs contributing to ECM regulation signatures at basal level in A549-WT or KI: Versican (VCAN), Fibroblast growth factor 5 (FGF5), Fibronectin Leucine Rich Transmembrane Protein 2 (FLRT2), CEA Cell Adhesion Molecule 6 (CEACAM6), Interleukin-6 (IL-6), and fibrinogen genes FGA, FGB, and FGG). Means ± SD of triplicates for two clones of each cell line are shown (*N* = 2). Statistical analyses were carried out as described in (**A**). Microarray values for the eight leading edge mRNAs (Fig. 4C) are shown in Supplementary Fig. 8. **D** Upper panel, Box plot of IL-6 protein levels as measured by SomaScan (SOMAmer anti_2573_20) in A549-WT and KI cells, at baseline and after a 24 h stimulation with P/I. Lower panel, Gene set enrichment analysis of longitudinal SomaScan data comparing the effects over time in A549-WT and KI cells. A selection of 124 gene sets related to ECM regulation was used. Shown are the 13 gene sets significant at a false discovery rate < 0.05; *NES* Normalized Enrichment Score.

We next turned our attention to factors produced by the cells that might influence the tumor environment. As prevention of CARD10 cleavage might sustain its signaling capacity, we stimulated A549-WT and KI cells with ligands known to trigger the C_10_BM pathway in various cell types, e.g., thrombin (THB), angiotensin II (AngII), LPS, EGF, and LPA^22^, and we measured several activation read outs, e.g., IFN, cytokines, chemokines as well as adhesion molecules. Most factors were not induced in A549 cells after stimulation except e.g., IL-8 (Supplementary Fig. 6). However, we noticed that basal levels of IL-6 were at least two-fold elevated in A549-KI cells, both at the mRNA and secreted protein levels, over the levels measured in A549-WT cells (Fig. 4A, Supplementary Fig. 7). In addition, IL-6 increased steadily upon P/I treatment and to higher levels in KI cells as compared to WT cells. Furthermore, upon TNF stimulation, IL-6 production in A549-KI and WT cells was similar at early time points but strongly increased in KI after 48 h reaching more than two-fold compared to WT cells at 72 h (Fig. 4A). Thus, IL-6 emerged as a factor sensitive to CARD10 cleavage, and data suggested that CARD10, although not directly involved in TNF signaling, might influence subsequent signaling events when CARD10 cleavage by MALT1 comes into play. Enhanced IL-6 production in A549-KI cells, whether at basal levels or after stimulation with P/I or TNF, was not abrogated by MALT1 protease inhibition, further suggesting that CARD10 cleavage impacts signaling beyond classical CBM mechanisms (Supplementary Fig. 7).

To obtain broader insights, we did an unbiased profiling at both mRNA and protein expression levels, in A549-WT and KI cells. Microarray transcriptomics data revealed 703 genes with significant differential expression in the KI clones, of which 480 were upregulated (Fig. 4B, upper panel). We then performed a gene set enrichment analysis using gene sets from multiple pathway collections (Fig. 4B, lower panel). The most significant pathways represented cancer and inflammation signaling as well as cancer-related processes, with a particular enrichment of gene sets linked to extra-cellular matrix regulation (ECM). A set of eight genes showing significant increase in expression (Supplementary Fig. 8), and often found across a number of these ECM-related pathways, were confirmed to be strongly upregulated in A549-KI cells by qPCR experiments (Fig. 4C). Next, we measured the expression levels of 4137 proteins in A549-WT and KI cells using the SomaScan^TM^, at baseline and after a 24 h stimulation with P/I. While no protein passed the strict thresholds for differential expression imposed by multiple hypothesis testing, IL-6 exhibited a trend of upregulation in KI compared to WT cells, both at baseline (fold-change 1.10, *p* value 0.31) and after stimulation (fold-change 1.18, *p* value 0.10) (Fig. 4D, upper panel). To confirm the enrichment in ECM components at the protein level, we conducted a gene set enrichment analysis focusing on 124 gene sets related to ECM regulation. Among these, 13 were significantly and positively enriched after P/I stimulation in KI as compared to WT cells (Fig. 4D, lower panel), further supporting a role for CARD10 cleavage by MALT1 in the regulation of extracellular matrix components.

Collectively, the data suggested a gain-of-function characterizing the non-cleavable form of CARD10, impacting exogenous factors and the regulation of the extracellular matrix, which might have accounted for the faster growth of A549-KI vs. WT tumors observed in the xenograft model.

## Discussion

Most known substrates of MALT1 are direct or indirect regulators of NF-κB, which illustrates the impact of MALT1 paracaspase activity on cellular responses after activation^48,49^. For example, A20, CYLD, RelB, Regnase, and Roquins^3,50,51^ have in common to be negative regulators of canonical NF-κB. However, HOIL1, another recently identified MALT1 substrate, participates in NF-κB activation^45,52^. In addition, BCL10 cleavage by MALT1 primes it for proteasomal degradation^28^ and C-terminal MALT1 auto-cleavage is inhibitory^25^, contributing to pathway down-regulation. Thus, the full outcome of MALT1 paracaspase activation is more complex than anticipated. All MALT1 substrates were initially described in lymphocytes where antigen receptor signaling triggers C_11_BM-dependent MALT1 activation. A substrate such as CYLD was subsequently shown to be cleaved in other cell types in the context of alternative CARD-CC signalosomes^53^. Expanding on this, herein we reported CARD10 as the first known non-hematopoietic MALT1 substrate, which adds further evidence for the broad regulatory impact of MALT1 paracaspase activity.

Cleavage of CARD10 was induced by P/I treatment of A549 cells, however it was also detectable in absence of any stimulation (Fig. 3A) suggesting a possible basal level of C_10_BM activation. Our laboratory previously reported that a PKC-dependent tonic CARD10 activation occurs in human primary keratinocytes^42^. Therefore, the body of evidence is growing that suggests some level of constitutive activity of CARD10, which clearly contrasts with the other CARD-CC proteins, which are locked in an auto-inhibitory state until they become activated^54^. It is therefore conceivable that CARD10 cleavage by MALT1 contributes a negative feedback mechanism that limits the signaling capacity under a threshold level. Regulation of IL-6 would support this hypothesis. IL-6 levels were almost undetectable in basal conditions in A549-WT cells. Preventing CARD10 cleavage was sufficient to raise IL-6 levels, as observed in various experimental settings with A549-KI cells, including a SomaScan.

IL-6 is often upregulated in cancers and is a key cytokine for tumorigenesis and metastasis^55^. Signaling through the IL-6 receptor strongly contributes to several indirect oncogenic processes such as invasion, inflammation, angiogenesis, and extracellular matrix modification^56^. Keeping IL-6 in check might be one important aspect of CARD10 cleavage by MALT1 and it might correlate, at least in part, with the extracellular matrix remodeling signature we found to be associated with CARD10 cleavage. Further experiments would be required to clarify whether IL-6 indeed has a leading role in this process, and more generally, to better understand primary *vs*. secondary signaling events downstream of CARD10 cleavage.

We showed that MALT1 cleaves CARD10 in the linker region, between the coiled-coil and the MAGUK domains. By removing the MAGUK domain, which is important for activity^31^, CARD10 cleavage by MALT1 likely reduces CARD10’s capacity to transduce signals at the membrane but it might also generate an intracellular CARD-CC moiety, reminiscent of CARD9. Cytoplasmic signaling of CARD-CC protein remains an understudied field, which would be worth further investigations. In that regard, a recent report showed that CARD10 contributes to the intracellular RIG-I/MAVS pathway^57^. In addition, several isoforms have been reported for the CARD-CC protein CARD14, among which shCARD14, missing the C-terminal part of the MAGUK domain, is believed to have an important function^58,59^.

The current understanding of CARD10 pathophysiological function in cancer is still limited. Studies were mostly conducted in vitro upon CARD10 overexpression or knock-down, and were essentially focused on proliferation readouts. In addition, work has been hampered by the low performance of available CARD10 antibodies, which remains a hurdle for upcoming investigations. It would be very interesting to evaluate tissue samples from CARD10 overexpressing tumors and measure the extent of CARD10 cleavage, which might inversely correlate with aggressiveness. The human COSMIC database mentions only two patients with an R587 mutation (COSM243479 and COSM1308145). However, mutations beyond the MALT1-cleavage site identified here might have an impact on the levels of CARD10 vs. its cleaved counterpart.

## Materials and methods

General information about cells, animals, antibodies and plasmids, as well as CRISPR, microarray, and SomaScan^TM^ procedures can be found in the Supplementary Information file.

### Ectopic expression in HEK293 cells

Cells were seeded at 0.15 x 10E6 cells/ well in 500 µl of DMEM 10% FCS in 24 well plates. On the next day, cells were transfected with 1 µg of total DNA (equally divided between all plasmid transfected and completed with empty vector according to conditions) using the 6:1 ratio of X-tremeGene^TM^ 9 DNA (Roche) according to the manufacturer’s instructions. Four hours later, cells were treated with compounds added in 500 µl of media with 2x compound concentration, with a final concentration of 0.33% of DMSO (Sigma, D2650). Cells were incubated for 24 h at 37 °C with 5% CO_2_. Cells were washed with PBS containing inhibitors (Phosphatase Inhibitor Cocktail 2 and 3; cOmplete™, EDTA-free Protease Inhibitor Cocktail, Roche) and lysed (Cell Signaling Technology^R^ 98035). Samples were denatured using the NuPAGE^TM^ LDS sample buffer 4X (Invitrogen) and NuPAGE^TM^ Sample Reducing Agent 10X (Invitrogen) and heated 10 min at 95 °C. Western blot was then performed using NuPAGE^TM^ 4–12% Bis-Tris Gels (Invitrogen) in MES SDS Running buffer (Invitrogen) and transfered using an i-blot^TM^ gel transfer device (Invitrogen i-blot^TM^ PVDF transfer stack regular). Membranes were blocked, incubated with antibodies and revealed using LI-COR Odyssey Infrared Imaging System (LI-COR Biosciences).

### NF-κB luciferase assay

Cells were trypsinized and diluted 1 to 2 in puromycin free media on the day before transfection. On the next day, cells were counted and set to 0.3 x 10E6 cells/ml and 1 µg DNA total/ml of cell was transfected in suspension according to manufacturer instruction using the 6:1 ratio of X-tremeGene^TM^ 9 DNA. For plasmid increasing doses, DNA quantity was completed with empty vector. Cells were then plated at 0.03 x 10E6 cells/per well in 100 µl of media in 96 well plate and incubated overnight at 37 °C, 5% CO_2_. 50 μl/ well of Britelite^TM^ plus reporter gene assay reagent (Perkin Elmer) pre-warmed at RT was added and luminescence was read using a Luminescence plate reader (EnVision, Perkin Elmer).

### Immunoprecipitation of endogenous CARD10

Cells (3 x 10E6) were plated in 10 cm petri dishes (3 dishes per condition). Four hours later, cells were treated with 1 µM of MLT-748 or DMSO and incubated overnight at 37 °C, 5% CO_2_. Cells were then treated with 3 μM of proteasome inhibitor MG-132 for 2 h and with PMA (50 µg/ml) + ionomycin (1 µM) for two additional hours. Cells were washed with PBS containing protease and phosphatase inhibitors (as above) and 80 μl of lysis buffer (Cell Signaling Technology 98035) was added directly on the plates kept on ice. Cells were scrapped, collected, and sonicated. Immunoprecipitation was performed using Dynabeads® Protein G Immunoprecipitation (Thermo Scientfic, 10007D) and samples were run on NuPAGE^TM^ 4–12% Bis-Tris Gels (Invitrogen). The anti-CARD10 primary antibody (abcam 137383) was used at 1/1000 dilution.

### A549 xenograft model in nude mice

A549-WT and KI clones 1 and 2 (obtained from two independent nucleotransfection rounds) were thawed and kept in culture for two passages in DMEM 10% FCS 1% L-Glutamine. Cells with 50% Matrigel (Corning) were s.c. injected in the right flank of 6–11 nude mice per group (specified in the legend of Fig. 3) at 2Mi cells per inoculation. Tumor growth was measured bi-weekly for 36–50 days using a caliper. After termination, tumors were collected, weighed, placed into 10% v/v neutral phosphate buffered formalin solution (Avantor) for 24 h for histology prior to tissue processing or snap-frozen in liquid nitrogen for further analysis. Formalin-fixed tumors were rinsed for 5 min under running tap water, dehydrated with graded ethanol concentrations (50% to 100%), cleared with xylene and infiltrated with paraffin overnight using a vacuum infiltration tissue processor (Leica Biosystems). Tumor tissues were then embedded in paraffin blocks. Paraffin sections were stained with haematoxylin and eosin (H&E). Staining for Ki67 was performed on a Ventana Discovery XT immunostainer (Roche Diagnostics), staining for msCD31 and SMA on a BondRX immunostainer (Leica Biosystems). Slides were digitalized using a ScanScope XT slide scanner (Leica Biosystems) with objective x20.

## Supplementary information

Supplementary Figures

Supplementary Materials and Methods

Rebuttal letter
