# Supplementary material for: CARD10 cleavage by MALT1 restricts lung carcinoma growth in vivo
Source: Oncogenesis. 2021 Apr 6;10(4):32. doi: 10.1038/s41389-021-00321-2 (PMC8024357; doi:10.1038/s41389-021-00321-2)
Supplement: Supplementary file 1 — Supplementary Figures [file 41389_2021_321_MOESM1_ESM.pdf]

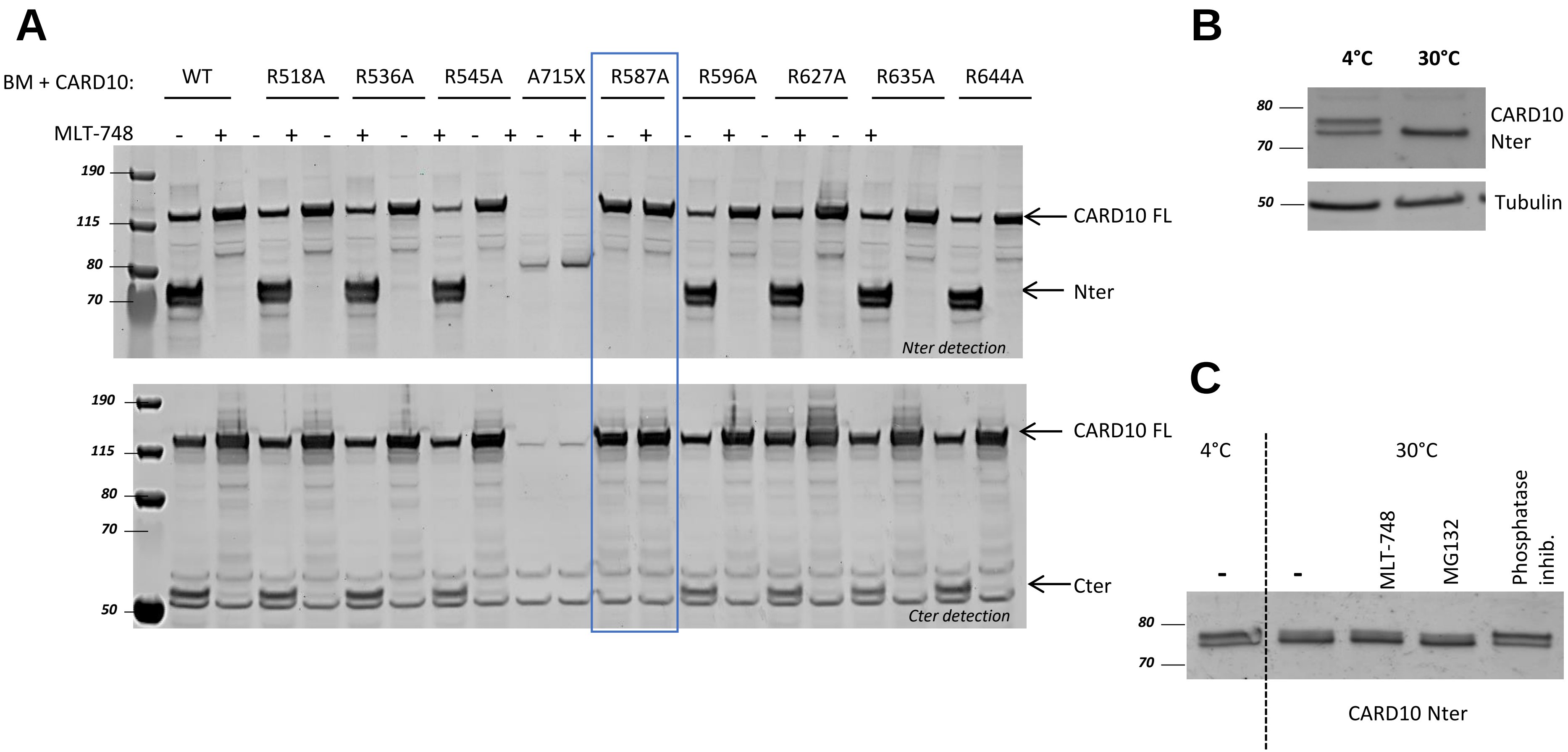

Supp Fig. 1: CARD10 cleavage site identification

(A) Site directed mutagenesis was performed on CARD10-WT expressing plasmid by swapping Arginine for Alanine residues as indicated. Each plasmid was transfected in HEK 293T cells together with MALT1 and BCL10 ± 1 µM of MLT-748. CARD10 expression was then assessed by Western blot using antibody with epitopes in the N-terminal (upper panel) or C-terminal (lower panel) part of the protein. **(B and C)** HEK 293T cells were transfected with C_{10}BM components and the observed CARD10-Nter doublet was characterized by incubating the whole cell extract with a MALT1 protease inhibitor (MLT-748, 1 µM), or proteasome inhibitor (MG-132, 5 µM) or a phosphatase inhibitor cocktail (2+3, see Methods) for 30 minutes at 30°C before Western blotting.

A

Human MALT1 substrates	Cleavage site					
		P4	P3	P2	P1	P+1
CARD10	WPLGKPEG	L	L	A	R	G
MALT1	NVTPADSC	H	C	S	R	T
MALT1	VLAGQFVK	L	C	C	R	A
HOIL-1	DLGFKDLT	L	Q	P	R	G
Roquin-2	NAENSVSQ	L	I	S	R	S
Roquin-1	STGNTVTQ	L	I	P	R	G
Regnase-1	PDPCPQLP	L	V	P	R	G
RELB	GPGEGLPR	L	V	S	R	G
CYLD	ERRPPKLA	F	M	S	R	G
A20	EGLPGMAL	G	A	S	R	G
BCL10	NSSEMFLP	L	R	S	R	T
**Consensus**		L	x	S/P	R	G

Sequence of MALT1 cleavage site in previously described substrates

B

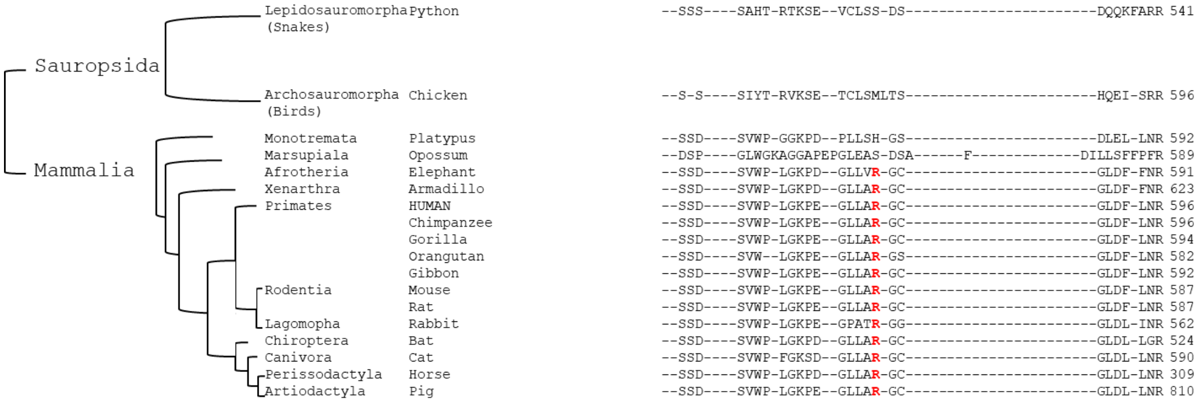

MALT1 R587 conservation in CARD10 vertebrate orthologues

Supp Fig. 2: CARD10 cleavage site conservation

(A) Sequence of MALT1 cleavage site in previously described human MALT1 substrates **(B)** MALT1 R587 conservation in CARD10 vertebrate orthologues (one species in each group was chosen as representative of the group).

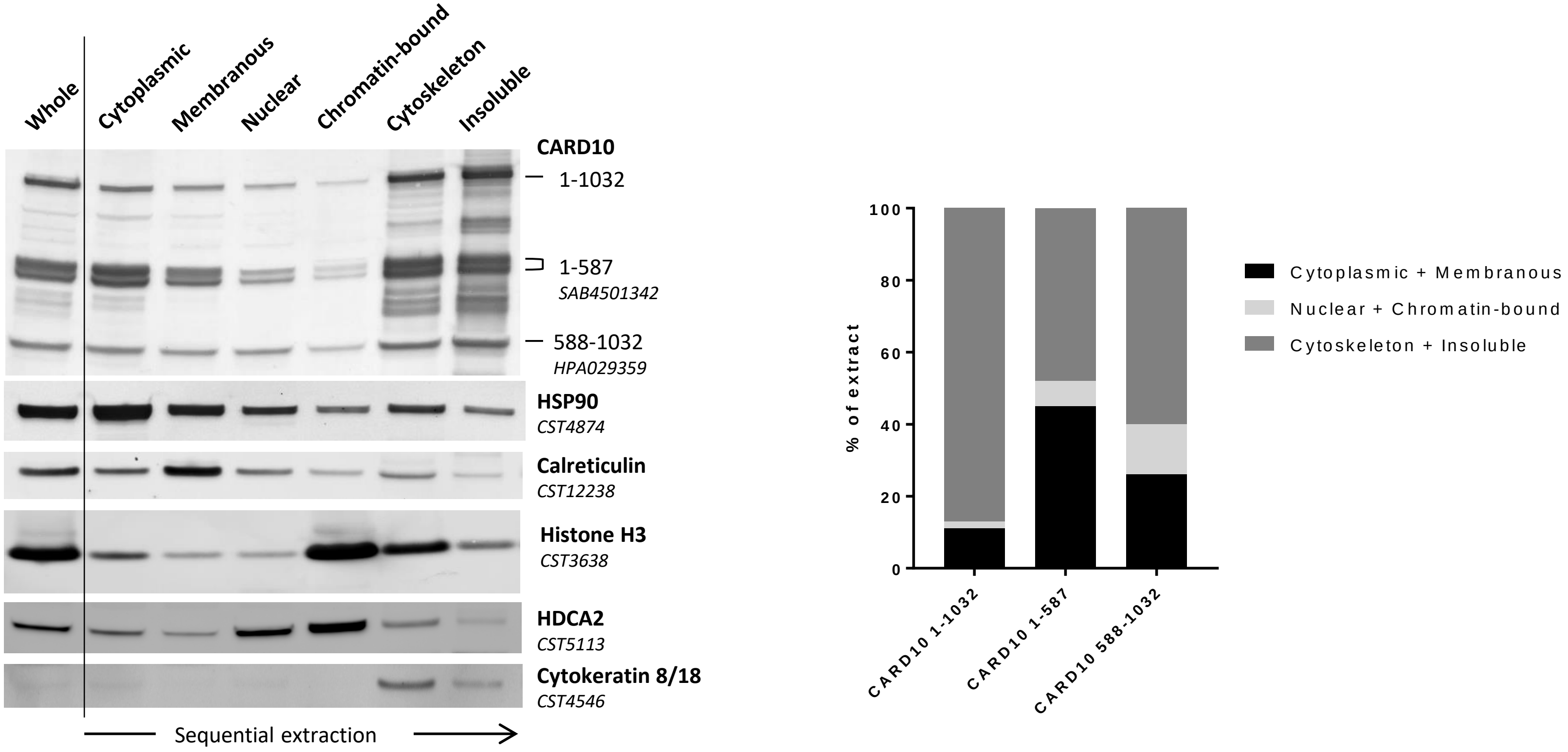

Supp Fig. 3: Cleavage of CARD10 by MALT1 releases an N-ter fragment with increased solubility

HEK 293T cells were transfected with C_{10}BM plasmid components and expressed proteins were subsequently extracted in a sequential manner using the Subcellular Protein Fractionation Kit for Cultured cells (Thermo Scientific, 78840). The content of CARD10 and fragments thereof in each fraction was assessed by Western blot using reference proteins. Relative enrichment is shown on the graph.

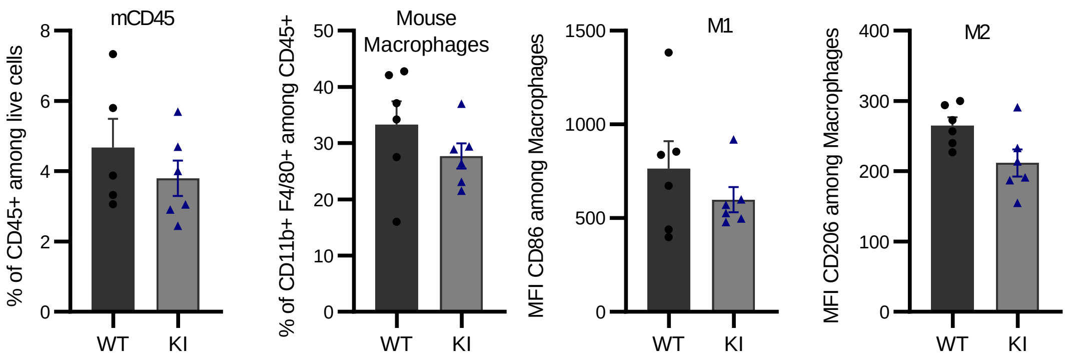

Supp Fig. 4: Tumor flow cytometry

Flow cytometry analysis of tumor cells after termination of the N1 experiment.

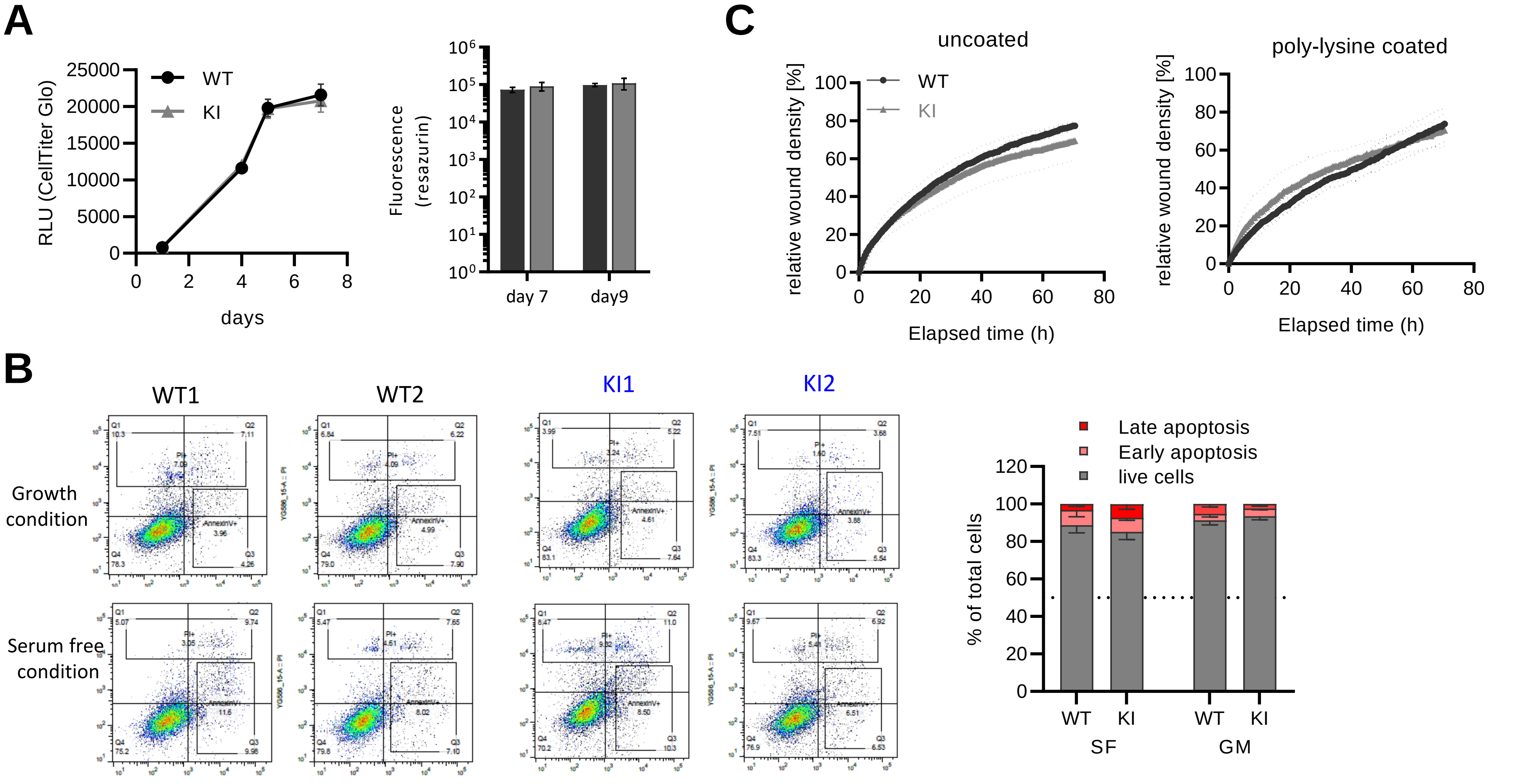

Supp Fig. 5: Preventing CARD10 cleavage has no impact on cell proliferation, apoptosis or migration

(A) Cells were plated at day 0 and cell proliferation was measured using Cell Titer Glo in A549-WT and KI lines (2 clones for each, 12 replicates for each clone) from 1 to 7 days in 96 well plates. Data represent means ± SD (left panel). Cells were plated at day 0 in media containing 2% agarose and proliferation was assessed at day 7 and day 9 using resazurin (right panel) **(B)** Cells were kept in culture for 3 days in growth media or serum free media and apoptosis was assessed by flow cytometry using Annexin V/PI staining. **(C)** Cells were plated at day 0 in complete media, in uncoated (left) or poly-lysine coated (right) plates, wells were scratched at day 1 (using IncuCyte wound maker) and imaged using the IncuCyte system for 3 days to measure wound closure.

RO / Stim	CXCL1 mRNA	ICAM1 Prot-FACs	IFNγ Prot-ELISA	IL-10 Prot-ELISA	IL12p70 Prot-ELISA	IL-13 Prot-ELISA	IL-1β Prot-ELISA	IL-2 Prot-ELISA	IL-4 Prot-ELISA	IL-6 Prot-ELISA	IL-8 Prot-ELISA	TNFα Prot-ELISA
**Basal**	Not induced	Not induced	Not induced	Not induced	Not induced	Not induced	Not induced	Not induced	Not induced	KI>WT (Fig 4A)	Not induced	Not induced
**P/I**	KI=WT	KI=WT	Not induced	Not induced	Not induced	KI=WT	Not induced	Not induced	Not induced	KI>WT (Fig 4A)	KI=WT	Not induced
**THB/TRAP6**	Not tested	KI=WT	Not induced	Not induced	Not induced	KI=WT	Not induced	Not induced	Not induced	Not induced	KI=WT	Not induced
**AngII**	Not tested	Not tested	Not induced	Not induced	Not induced	Not induced	Not induced	Not induced	Not induced	Not induced	KI=WT	Not induced
**LPS**	Not tested	Not tested	Not induced	Not induced	Not induced	Not induced	Not induced	Not induced	Not induced	Not induced	KI=WT	Not induced
**EGF**	Not tested	Not tested	Not induced	Not induced	Not induced	Not induced	Not induced	Not induced	Not induced	Not induced	KI=WT	Not induced
**LPA**	Not tested	Not tested	Not induced	Not induced	Not induced	Not induced	Not induced	Not induced	Not induced	Not induced	KI=WT	Not induced
**TNFα**	KI=WT	KI=WT	Not induced	KI=WT	Not induced	KI=WT	Not induced	Not induced	Not induced	KI>WT (Fig 4A)	KI=WT	

Supp Fig. 6: Read outs measured in A549-WT *vs* KI cells

Table showing read-outs measured upon various stimulatory conditions

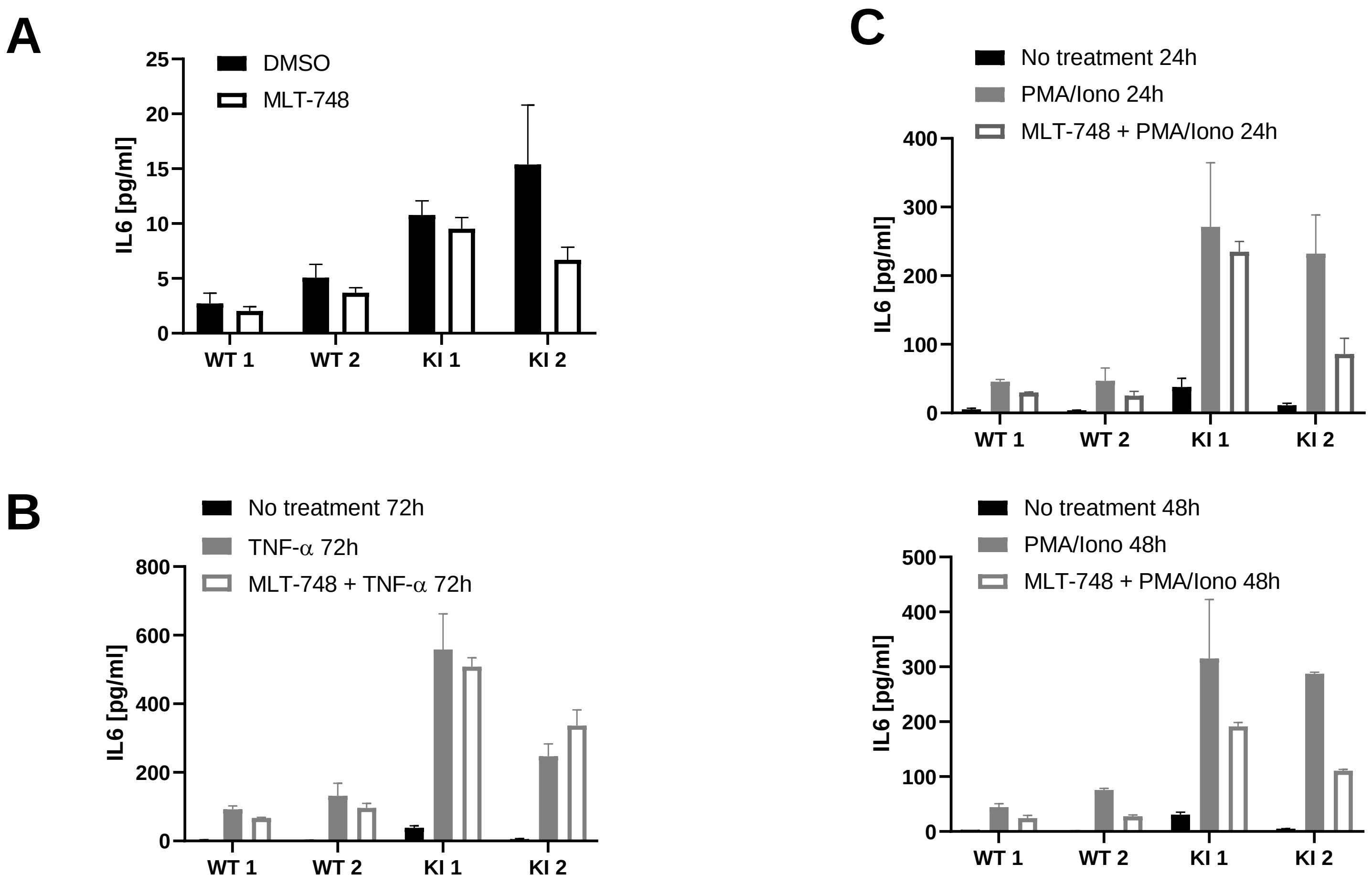

Supp Fig. 7: MALT1 protease inhibition does not normalize the enhanced IL-6 levels in A549-KI cells

(A) 10’000 A549 cells/well in 6 replicates in 384-well plate. Treatment with vehicle (DMSO) or 1 µM MLT-748 for 48h. IL-6 levels in supernatants measured by MSD. **(B)**,**(C)** 30’000 A549-WT or A549-KI cells/well in triplicates in 384-well plate. Pretreatment with vehicle (DMSO) or 1 µM MLT-748 for 1 hour. Stimulate with 50 ng/ml PMA/1 µM Ionomycin for 24h and 48h **(B)**, or with 10 ng/ml TNF-α for 72h **(C)**. IL-6 levels in supernatants measured by MSD. All data presented as mean +SD.

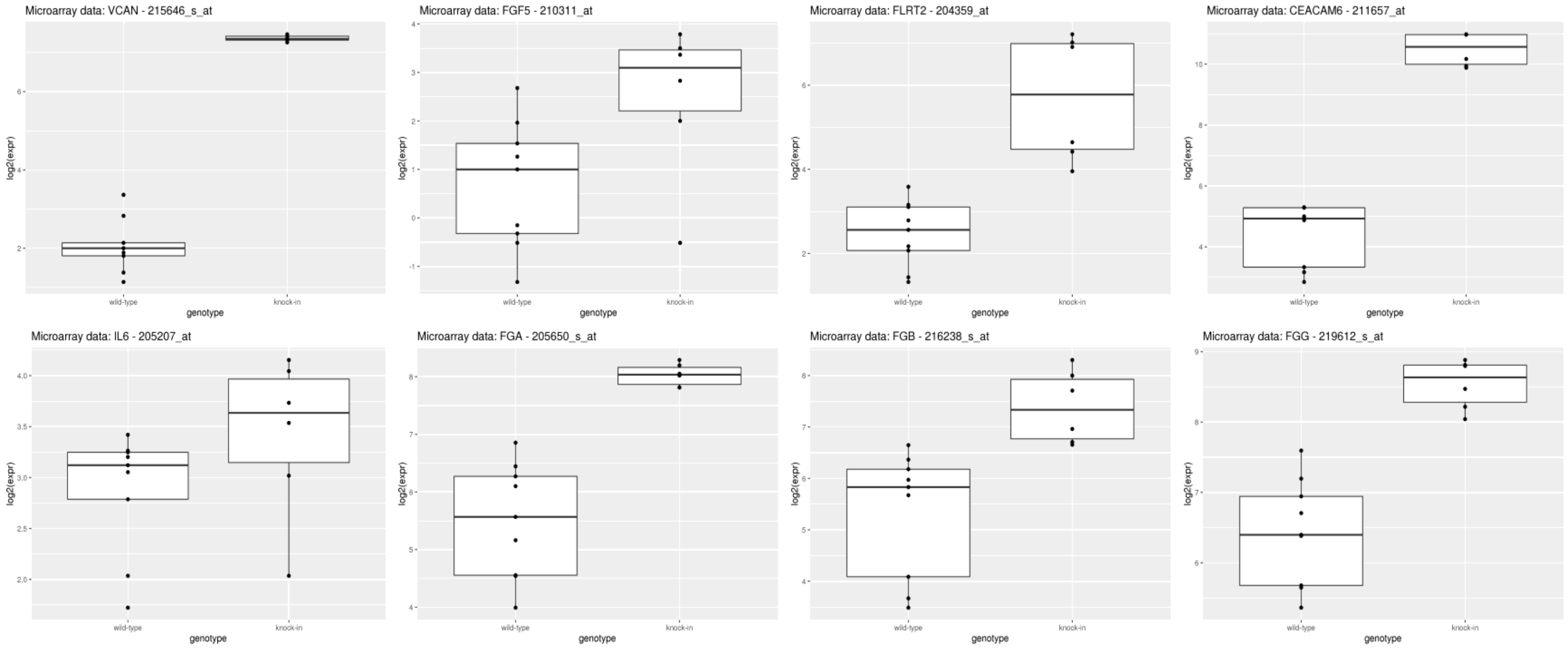

Supp Fig. 8: Preventing CARD10 cleavage deregulates ECM components

Box plots of eight selected mRNAs as measured by microarray in A549- WT and KI cells at baseline.
