# Supplementary material for: CARD10 cleavage by MALT1 restricts lung carcinoma growth in vivo
Source: Oncogenesis. 2021 Apr 6;10(4):32. doi: 10.1038/s41389-021-00321-2 (PMC8024357; doi:10.1038/s41389-021-00321-2)
Supplement: Supplementary file 2 — Supplementary Materials and Methods [file 41389_2021_321_MOESM2_ESM.pdf]

Supplementary Materials and Methods section

Cells and animals

HEK 293T cells (CRL-11268) and A549 (CCL-185) were purchased from ATCC and cultured in DMEM High glucose No Glutamine (1960-044 GIBCO) supplemented with 10% FBS Gold FCS (Heat inactivated PAA #A15-152 lot #A15211-0991). HEK-NFκB luciferase reporter cells were maintained in DMEM 10% FCS containing puromycin (Sigma, 9620). HuVECs were acquired from PromoCell (C-12203). A549 cell lines were monthly tested for mycoplasma.

All animal experiments were performed according to procedures covered by permit number BS-2808 issued by the Kantonales Veterinäramt Basel-Stadt and strictly adhered to the Eidgenössisches Tierschutzgesetz and the Eidgenössische Tierschutzverordnung. Female athymic nude (nu/nu) mice were obtained from Charles Rivers (Germany). All the animals were housed in a pathogen-controlled environment with access to food and water *ad libitum* and they were identified with transponders.

Antibodies and plasmids

Primary antibodies CARD9 (12416), CARD11 (4435), CYLD (8462), RelB (D1A10) were purchased from Cell Signaling Technology (Massachusetts). MALT1 (MT1/410), BCL10 (EP606Y), CARD10 (Nter detection in A549 cells - ab137383) and CARD14 (ab64366), SMA (EPR5368) were purchased from Abcam (Cambridge, UK). CARD10 (Nter detection in HEK cells SAB 4501342) or CARD10 (C-ter detection HPA029359), HOIL1 (HPA024185), Tubulin-α (T6074) were purchased from Sigma-Aldrich (Missouri). Primary antibodies were used 1/1000 diluted. Secondary antibody goat anti-rabbit IRDye800CW (LI-COR Biosciences 926-32211) and goat anti-mouse IgG (H+L) Alexa Fluor 680-F(ab')2 fragment (Molecular Probe A21059) were used 1/10000 diluted. Primary antibodies for IHC Ki67 (SP6) was purchased from NeoMarkers and msCD31 (SZ31) from Dianova. Secondary antibodies for IHC goat anti-rabbit

biotinylated was purchased from Jackson ImmunoResearch and rabbit anti-rat with Polymer Refine detection from Leica Biosystems.

MALT1, BC10 and CARD11 plasmids were generated internally. CARD9 (NM_052814.3), CARD10 (NM_014550.3), CARD14 (NM_024110.4) and CYLD (NM_015247.2) were purchased from Genecopoeia. CARD10 mutagenesis was performed according to the manufacturer instruction (QuikChange II XL Kit, Agilent Techology, 200521-5). CARD10-R587A was generated with the following primers containing the Arg to Ala change (R587A F: GGAAGGCCTCCTGGCTGCCGGCTGTGGCCT and R587A R: AGGCCACAGCCGGCAGCCAGGAGGCCTTCC); CARD10-Nter (G588X) by inserting a STOP codon at position 588 (F: CCGGAAGGCCTCCTGGCTCGGTAATGTTAACTGGACTTCCTCAACAGGTCT, R: AGACCTGTTGAGGAAGTCCAGTTAACATTACCGAGCCAGGAGGCCTTCCGG) and CARD10-Cter (588-1032) by inserting a large deletion (F: GAATTCGGTACCATGGGCTGTGGCCTGGAC, R: GTCCAGGCCACAGCCCATGGTACCGAATTC).

CRISPR

CRISPR knock-in was performed on the parental A549 cell line from ATCC at passage 4 after purchase. Cells were treated for 5 hours with 5 µM of the DNA-PK inhibitor NU-7441 (Novartis) to reduce the frequency of NHEJ and increase the efficiency of homology-directed repair (HDR)[1]. Three millions cells were then nucleotransfected with the Neon system (Thermofisher) according to manufacturer instruction with the following mix in 100 µl total volume : (i) 2 µM of CARD10 gRNA (CCGGAAGGCCTCCTGGCTCG) with ATTO550 fluorescent tracrRNA (IDT DNA) located in exon 11 close to the R587 site, (ii) 2 µM of the repair template (CAT CCT CTG ACA GCG TGT GGC CTT TGG GAA AGC CGG AAG GCC TCC TGG CAG CCG GCT GTG GCC TGG ACT TCC TCA ACA GGT AC) (Microsynth) containing the Ala587 mutation as well as new restriction sites based on codon degeneracy for genotyping, (iii) 0.2

µg/µl of CAS9-GFP (Novartis). Incubate cells 24 hours at 37°C, 5%CO_2 in conditioned media and sort single clones in 96 well plates based on double positive population (gRNA – ATTO550 and Cas9-GFP). Monitor clone growth and image plates every 2-3 days to identify single clones. For each confluent well, PCR was performed on CARD10 exon 11 (F: GGCCTCAAACCTGCCAAGG and R: CCATGCAAAAAGGGTCATCATCTCC) and digestion with AvaI (present in WT sequence) and NaeI (modified in edited sequence). NaeI digested/AvaI non-digested clones were then confirmed by sequencing of exon 11 of CARD10.

Microarray

mRNA from A549 clones WT and KI-CRISPR modified was extracted using RNeasy Mini Kit (Qiagen, 74104) and dosed using Nanodrop. The extracted mRNA samples were hybridized to Affymetrix Human Genome U133 Plus 2.0 microarrays. Hybridization, washing and staining of the microarrays were done according to Affymetrix standard protocols. The arrays were scanned using an Agilent Technologies GeneChip Scanner 3000 (Agilent Technologies), and the images converted to expression values using the Affymetrix Microarray Analysis Suite 5.0 software. Further data analysis was done on per chip normalized expression values using version 3.6.1 of the R scripting language. Differential expression analysis was done with the limma R package[2] (version 3.42.2) using the formula "~ 0 + genotype + clone + (1|replicate)" where genotype refers to WT or KI, clone to one of three WT clones or two KI clones, and replicate to the sample triplicates. Multiple testing correction was done using the Benjamini-Hochberg false discovery rate.

For gene set enrichment analysis of the microarray data, the R package fgsea was used[3]. In cases where a gene was represented by more than one probe set, the probe set with the best t score was used. As gene sets, the MSigDB version 7.0.1 collections hallmark, C2-CP, C2-BIOCARTA, C2-KEGG, C2-PID, C2-

REACTOME and the MetaBase version 19.4.69900 collection “maps” were used[4–6] (see http://software.broadinstitute.org/gsea/msigdb/collections.jsp for a description of the MSigDB collections, and https://www.gsea-msigdb.org/gsea/msigdb/collection_details.jsp for their references; MetaBase is a commercial product of Clarivate). The analysis was performed over 100,000 permutations with gene sets having at least 10 and not more than 1,000 genes.

SomaScan™

A549 clones were plated at 0.3 x10E6 cells per well in 6 well plates in DMEM 10% FCS. On the next day, cells were stimulated in fresh media with PMA (50 μg/ml) plus ionomycin (1 μM) for 24 hours. Cells were then washed with PBS and lysed using M-PER Mammalian Protein Extraction Reagent (Thermo Scientific) containing HALT protease inhibitors. Protein quantification was performed (Quick Start™ Bradford Protein Assay Kit, Biorad, 5000201) and samples were diluted to 0.2 mg/ml, stored at −80 °C and shipped to SomaLogic (Boulder, CO) for SomaScan. In total, the expressions levels of 4,137 distinct human gene targets (as measured by 4,783 SOMAmers recognizing human proteins) were determined in an expanded platform[7] with a method similar to the earlier version 2 of the assay detailed elsewhere[8].

Differential protein expression analysis was also done with the limma R package[2] (version 3.42.2), using the formula "~ 0 + genotype_stimulation + clone" where genotype_stimulation refers to the combination of genotype (WT or KI) and stimulation (baseline or 24 hours of stimulation with P/I), and clone refers to one of two WT or KI clones. Multiple testing correction was done using the Benjamini-Hochberg false discovery rate with a cutoff of < 0.05.

Gene set enrichment analysis of the SomaScan data was done with the R package fgsea[3] (version 1.12.0) and using the SOMAmer^{R} protein with the best t score when a gene was represented by more than one SOMAmer. The same MSigDB and MetaBase gene set collections described above were used, with the

addition of MSigDB C5-BP. These were filtered for ECM-related gene sets by using the keywords “cell adhesion”, “ECM”, “extracellular matrix”, “matrisome” and “integrin”, resulting in 124 gene sets. Furthermore, to adjust for the smaller universe of proteins covered by the SomaScan platform, the selected gene sets were filtered to contain only genes encoding for proteins covered in the SomaScan platform. The analysis was performed over 100,000 permutations with gene sets having at least 10 and not more than 1,000 genes.

1 Robert F, Barbeau M, Éthier S, Dostie J, Pelletier J. Pharmacological inhibition of DNA-PK stimulates Cas9-mediated genome editing. *Genome Med* 2015. doi:10.1186/s13073-015-0215-6.

2 Smyth GK. Linear models and empirical bayes methods for assessing differential expression in microarray experiments. *Stat Appl Genet Mol Biol* 2004. doi:10.2202/1544-6115.1027.

3 Sergushichev AA. An algorithm for fast preranked gene set enrichment analysis using cumulative statistic calculation. *bioRxiv* 2016. doi:10.1101/060012.

4 Subramanian A, Tamayo P, Mootha VK, Mukherjee S, Ebert BL, Gillette MA *et al.* Gene set enrichment analysis: A knowledge-based approach for interpreting genome-wide expression profiles. *Proc Natl Acad Sci U S A* 2005. doi:10.1073/pnas.0506580102.

5 Liberzon A, Subramanian A, Pinchback R, Thorvaldsdóttir H, Tamayo P, Mesirov JP. Molecular signatures database (MSigDB) 3.0. *Bioinformatics* 2011. doi:10.1093/bioinformatics/btr260.

6 Liberzon A, Birger C, Thorvaldsdóttir H, Ghandi M, Mesirov JP, Tamayo P. The Molecular Signatures Database Hallmark Gene Set Collection. *Cell Syst* 2015. doi:10.1016/j.cels.2015.12.004.

7 Emilsson V, Ilkov M, Lamb JR, Finkel N, Gudmundsson EF, Pitts R *et al.* Co-regulatory networks of human serum proteins link genetics to disease. *Science (80-)* 2018. doi:10.1126/science.aaq1327.

8 Gold L, Ayers D, Bertino J, Bock C, Bock A, Brody EN *et al.* Aptamer-based multiplexed proteomic technology for biomarker discovery. *PLoS One* 2010. doi:10.1371/journal.pone.0015004.
