# Supplementary material for: CARD10 cleavage by MALT1 restricts lung carcinoma growth in vivo
Source: Oncogenesis. 2021 Apr 6;10(4):32. doi: 10.1038/s41389-021-00321-2 (PMC8024357; doi:10.1038/s41389-021-00321-2)
Supplement: Supplementary file 3 — Rebuttal letter [file 41389_2021_321_MOESM3_ESM.pdf]

Reviewer #1 (Comments to the Author):

In this manuscript, authors suggest MALT1 modulates CARD10 activity through proteolytic cleavage of the protein. They also show that an un-cleavable mutant of CARD10 promotes tumor growth in a mouse xenograft model and enhances IL-6 production.
The manuscript has already undergone a cycle of peer-review, and the authors have improved their work by showing that MALT1-mediated cleavage of CARD10 occurs constitutively in cultured cells. This data is consistent with what occurs for CARD14 which, through alternative splicing, constitutively expresses proteic forms very similar to those generated through the MALT1-promoted cleavage of CARD10.
Although the authors do not clarify the molecular mechanism by which the CARD10 mutant resistant to MALT1 proteolytic cleavage promotes tumor growth and increases IL6 expression level, this reviewer thinks that the data generated are still important and deserve publication.

Reviewer #2 (Comments to the Author):

The authors report on a novel cleavage event of CARD10, which seems to be a negative feedback loop by generating a less active CARD9-like N terminal fragment of CARD10. These truncated forms still signal to NF-kB and this raises the fundamental question whether the subcellular localization of CARD9 and cleaved CARD10 vs the other CARMA members of the CARD-CC family also influence other aspects of the signaling and response in the cell. Could it be that cytoplasmic cleaved CARD10 is a also a gain-of-function fragment that triggers alternative responses?

- *This is an excellent point. CARD9 and now cleaved CARD10 too, have distinct properties compared to their MAGUK-containing CC-CARD homologs. Addressing whether they might contribute to intracellular signaling pathways will be highly interesting to pursue. In this regard, a soluble form of Dectin-1 (Dectin -1E) was previously identified, which remains to be characterized.*

Further, they demonstrate that this cleavage in non-hemapoetic cells may be an important cancer-suppressing function of MALT1 protease activity by down-regulating over-active CARD10-dependent signaling.

This finding is highly intriguing and makes a lot of sense considering that the other two components of the CBM complex have been shown to be cleaved: Bcl10 and MALT1 itself (at 2 sites). I am surprised that this has not been seen/reported earlier, especially considering how dramatic the effect is by coexpression of all 3 components (Fig 1A).

- *Just a short note here: Co-expression of the 3 CBM components has not been widely used in fact. In several studies, MALT1 is co-expressed only with BCL10, which can be sufficient to stimulate MALT1 protease function.*

Minor comments

line 133: reference 39 has a strange author list formatting. It might be interesting to look at PMID: 32790937

The kappa symbol replaced by "??" in several references

- *We have thoroughly checked the references in the revised version.*

line 156+ : The use of KI clones is of course an elegant way of avoiding artifacts from overexpression, but also carries its own risk. How did you ensure that the two different clones were in fact independent clones? Line 477 indicate 24h culture after transfection before single cell sorting. Are 2 clones enough to avoid the risk of artifact results from clonal variation?

- *The two clones used were derived from two independent transfection experiments. We have now added this note in the Methods section.*

It is intriguing that uncleavable CARD10 does not lead to higher NF-kB activation but does increase IL-6 expression. The IL-6 mRNA is one that is sensitive to post-transcriptional destabilization by MALT1 protease-sensitive proteins (roquin-1 mutants, for example, show high IL-6 expression). Could the increase of IL-6 be due to higher protease activity (or perhaps localization-dependent protease activity)? It would be interesting to compare IL-6 production in WT and KI cells in presence of MALT1 protease inhibitor.

- *We have performed additional experiments with the MALT1 protease inhibitor MLT-748, showing that MALT1 protease function is not responsible for the higher basal and stimulated (PMA, TNF) IL-6 levels in A549-KI cells. We have now summarized these data in a new supplementary figure (Supp Fig. 7) and added a sentence in the text.*

Along the same lines, it might be interesting to see how many of the differentially expressed genes that were found in the comparisons between WT and KI are in fact regulated by MALT1 protease-sensitive mRNA destabilizing proteins.

- *We checked the RNA levels of Roquins 1 and 2 as well as Regnase-1, but there was no difference between A549-WT and A549-KI cells.*

Since I have been recruited at a later stage in the review process, I will also comment on the response to the previous reviewers.

The responses to reviewer 1 seem satisfactory. Especially the important point that the KI cell lines do show a relevance for the cleavage at endogenous levels in some cancer cells.

The reviewer 2 questions 2&3 overlap with my proposal above with enhanced MALT1 protease activity leading to elevated IL-6 mRNA stability as a possible mechanism. This could be a very easy experiment to do and would in fact be in scope of this article, especially if this

indeed is the mechanism. The important point would be: Does the difference between WT and KI cells in IL-6 production disappear in presence of MALT1 inhibitor?

➢ *As mentioned above, the difference between A549-WT and A549-KI cells does not disappear in the presence of a MALT1 inhibitor (Supplementary Figure 7 recapitulates these new data).*

Answers to reviewer 2 question 4 satisfies my question above regarding sufficient number of clones.

Reviewer 2 minor point 1 : LLAR/G is in fact a much better substrate profile than many of the other known substrate sites.

➢ *Indeed. We have added a sentence in the text and have provided the table below as Supplementary Figure 2A.*

Human MALT1 substrates	Cleavage site					
		P4	P3	P2	P1	P+1
CARD10	WPLGKPEG	**L**	**L**	**A**	**R**	**G**
MALT1	NVTPADSC	**H**	**C**	**S**	**R**	**T**
MALT1	VLAGQFVK	**L**	**C**	**C**	**R**	**A**
HOIL-1	DLGFKDLT	**L**	**Q**	**P**	**R**	**G**
Roquin-2	NAENSVSQ	**L**	**I**	**S**	**R**	**S**
Roquin-1	STGNTVTQ	**L**	**I**	**P**	**R**	**G**
Regnase-1	PDPCPQLP	**L**	**V**	**P**	**R**	**G**
RELB	GPGEGLPR	**L**	**V**	**S**	**R**	**G**
CYLD	ERRPPKLA	**F**	**M**	**S**	**R**	**G**
A20	EGLPGMAL	**G**	**A**	**S**	**R**	**G**
BCL10	NSSEMFLP	**L**	**R**	**S**	**R**	**T**
**Consensus**		**L**	**x**	**S/P**	**R**	**G**

- The multiple sequence alignment only includes mammalian species. A brief check on a broader alignment on the entire CARD-CC family from the last common CARD-CC ancestor in tunicates to the four modern CARD-CC isoforms found in jawed vertebrates indicated that the cleavage site was absent in chicken and shark CARD10. The CARD10 cleavage might thus be a relatively recent “invention” specific to mammals.

➢ *This is a valuable suggestion. We have added a sentence in the text and have provided the alignments below as Supplementary Figure 2B.*

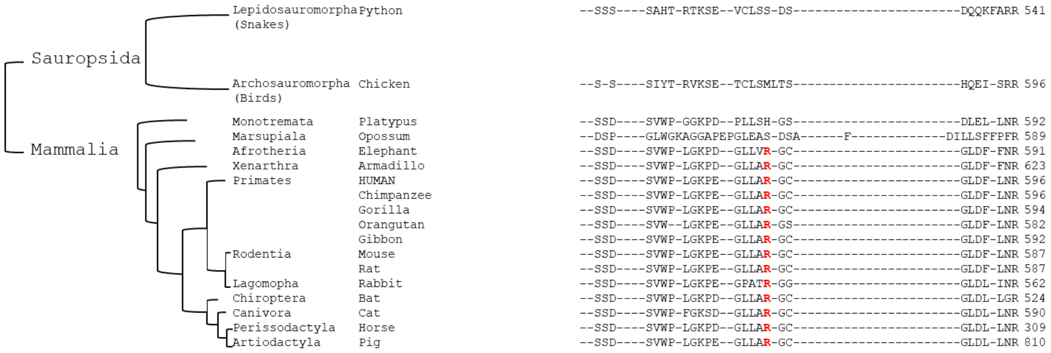
Sauropsida
Mammalia
Lepidosauromorpha (Snakes) Python --SSS----SAHT-RTKSE--VCLSS-DS-----------------------DQQKFARR 541
Archosauromorpha (Birds) Chicken --S-S----SIYT-RVKSE--TCLSMLTS-----------------------HQEI-SRR 596
Monotremata Platypus --SSD----SVWP-GGKPD--PLLSH-GS-----------------------DLEL-LNR 592
Marsupiala Opossum --DSP----GLWGKAGGAPEPGLEAS-DSA------F-------------DILLSFFPFR 589
Afrotheria Elephant --SSD----SVWP-LGKPD--GLLV**R**-GC-----------------------GLDF-FNR 591
Xenarthra Armadillo --SSD----SVWP-LGKPD--GLLA**R**-GC-----------------------GLDF-FNR 623
Primates HUMAN --SSD----SVWP-LGKPE--GLLA**R**-GC-----------------------GLDF-LNR 596
Chimpanzee --SSD----SVWP-LGKPE--GLLA**R**-GC-----------------------GLDF-LNR 596
Gorilla --SSD----SVWP-LGKPE--GLLA**R**-GC-----------------------GLDF-LNR 594
Orangutan --SSD----SVW--LGKPE--GLLA**R**-GS-----------------------GLDF-LNR 582
Gibbon --SSD----SVWP-LGKPE--GLLA**R**-GC-----------------------GLDF-LNR 592
Rodentia Mouse --SSD----SVWP-LGKPE--GLLA**R**-GC-----------------------GLDF-LNR 587
Rat --SSD----SVWP-LGKPE--GLLA**R**-GC-----------------------GLDF-LNR 587
Lagomopha Rabbit --SSD----SVWP-LGKPE--GPAT**R**-GG-----------------------GLDL-INR 562
Chiroptera Bat --SSD----SVWP-LGKPD--GLLA**R**-GC-----------------------GLDL-LGR 524
Canivora Cat --SSD----SVWP-FGKSD--GLLA**R**-GC-----------------------GLDL-LNR 590
Perissodactyla Horse --SSD----SVWP-LGKPD--GLLA**R**-GC-----------------------GLDL-LNR 309
Artiodactyla Pig --SSD----SVWP-LGKPE--GLLA**R**-GC-----------------------GLDL-LNR 810
